# Simple Fabrication of Solid-State Nanopores on a Carbon Film

**DOI:** 10.3390/mi12091135

**Published:** 2021-09-21

**Authors:** Natsumi Takai, Kan Shoji, Tei Maki, Ryuji Kawano

**Affiliations:** 1 Department of Biotechnology and Life Science, Tokyo University of Agriculture and Technology, Tokyo 184-8588, Japan; takai723nt@gmail.com (N.T.); tmaki@jeol.co.jp (T.M.); 2 Department of Mechanical Engineering, Nagaoka University of Technology, Niigata 940-2188, Japan; 3 JEOL Ltd., Tokyo 196-8558, Japan

**Keywords:** nanopore, solid-state nanopore, carbon thin film, dielectric breakdown

## Abstract

Solid-state nanopores are widely used as a platform for stochastic nanopore sensing because they can provide better robustness, controllable pore size, and higher integrability than biological nanopores. However, the fabrication procedures, including thin film preparation and nanopore formation, require advanced micro-and nano-fabrication techniques. Here, we describe the simple fabrication of solid-state nanopores in a commercially available material: a flat thin carbon film-coated micro-grid for a transmission electron microscope (TEM). We attempted two general methods for nanopore fabrication in the carbon film. The first method was a scanning TEM (STEM) electron beam method. Nanopores were fabricated by irradiating a focused electron beam on the carbon membrane on micro-grids, resulting in the production of nanopores with pore diameters ranging from 2 to 135 nm. The second attempt was a dielectric breakdown method. In this method, nanopores were fabricated by applying a transmembrane voltage of 10 or 30 V through the carbon film on micro-grids. As a result, nanopores with pore diameters ranging from 3.7 to 1345 nm were obtained. Since these nanopores were successfully fabricated in the commercially available carbon thin film using readily available devices, we believe that these solid-state nanopores offer great utility in the field of nanopore research.

## 1. Introduction

Nanopore technology has received extensive attention due to its unique capabilities on the single-molecule scale, which makes it useful for several applications: single-molecule detection [1,2,3,4], nanopore sequencing [5,6], and as nanopore filters [7,8]. With nanopore technology, there are two different types of nanopores, biological and solid-state nanopores. Biological nanopores are peptide- and protein-based nanopores and are reconstituted into a bilayer lipid membrane. Because biological nanopores can provide a much smaller pore size and higher spatial resolution compared with solid-state nanopores (ssNPs), they have already been applied as DNA sequencers [1,6,9,10,11,12,13,14,15]. On the other hand, glass nanopores [16], track-etched nanopores [17], and ssNPs [18,19] are constructed entirely of artificial materials. Because they have advantages over biological nanopores including controllability of pore size, and mechanical, electrical, thermal, and chemical stability, they are expected to be widely applied as the nanopore sensors with the highest durability.

The fabrication procedures and characteristics of these nanopores are as follows. Glass nanopores are formed by pulling glass capillaries with a pipette puller. They are widely used because they use the cheapest material (under $1 per single glass capillary) and the simplest fabrication procedure. By contrast, the effective length of these nanopores is longer than ssNPs, and the spatial resolution tends to be lower than other nanopores. Track-etched nanopores are fabricated by initially irradiating an ion beam on polymer membranes to form damaged tracks and subsequently etching the tracks. In this method, various geometries of nanopores can be fabricated by changing the tracking process and etching methods. However, because a heavy ion accelerator is required to form the damaged tracks, the fabrication process is quite costly. A single ssNP can be fabricated in a free-standing thin membrane by irradiation with ion/electron beam sculpting [20,21], a focused electron beam (EB) with a scanning transmission electron microscope (STEM) [22,23], or using dielectric breakdown (DB) [24,25,26]. These fabrication methods have already been established for various materials, such as silicon nitride (SiNx) [27,28] and silicon oxide (SiOx) [22,29], alumina materials [30,31], and graphene [32,33,34] or other 2-D materials [35,36]. However, before forming a single pore, an ultrathin membrane needs to be prepared. To fabricate such thin membranes, micro-and nano-fabrication techniques, including photolithography, chemical vapor deposition, and reactive ion etching are performed, but these fabrication procedures are usually time-consuming and costly. Therefore, a strategy for the simple preparation of the thin membrane at low cost is required for general research use and would facilitate the expansion of nanopore research. 

**Figure 1:**
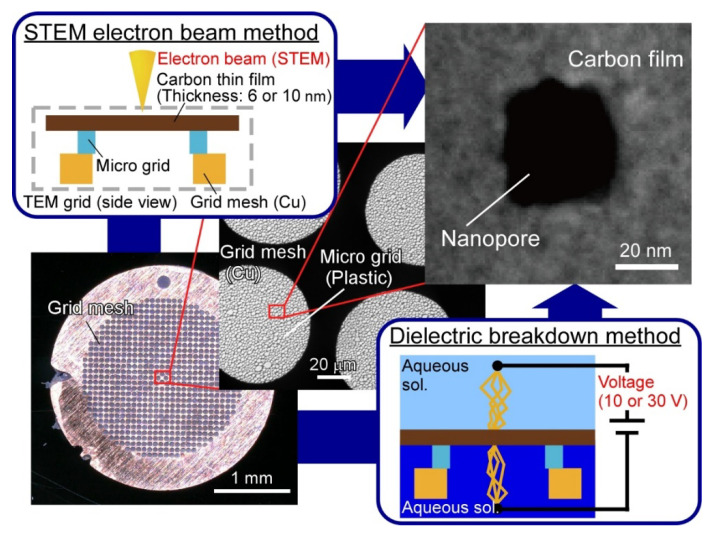
Schematic illustrations of strategies to fabricate solid-state nanopores in a carbon film on a micro-grid. The nanopores were fabricated by two different methods. One is the “STEM electron beam method”. In this method, the nanopores were fabricated using a STEM, by irradiating a focused electron beam on the carbon thin film. The other is the “dielectric breakdown method”. In this method, nanopores were formed by applying a trans-membrane voltage through the carbon thin film immersed in an electrolyte solution.

Herein, we propose the use of a commercially available thin carbon film coated on a micro-grid for TME observation as the thin membrane (Figure 1). The carbon membrane has a thickness of around 6 or 10 nm and is readily purchasable from various companies. In addition, carbon membrane with a 2 nm thickness can be purchased from JEOL Ltd., Japan, and nanopores with shorter pore lengths than conventional SINx and SiOx nanopores could potentially be fabricated. Besides, they are available at a reasonable price from several companies (around $10 per single micro-grid). Also, because the carbon film is on the micro-grid for TEM observation, the purchased membrane can be directly applied for nanopore fabrication by the STEM EB method. In addition, the possibility of surface modification and the functionalization of carbon [37] could offer specific sensing [38,39] and signal amplification [40]. We fabricated nanopores in the thin carbon membrane by either the STEM EB method or the DB method (Figure 1). In this paper, we discuss the simplicity and size-controllability of nanopores fabricated by these two methods by comparing them with conventional methods to fabricate solid-state nanopores. 

## 2. Materials and Methods

### 2.1. Experimental Materials

Micro-grids consisting of an amorphous carbon thin film coating with a thickness of 6 or 10 nm were purchased from Okenshoji Co., Ltd., Tokyo, Japan, or JEOL Ltd., Tokyo, Japan. The product No. of each grid is UHR-C10, SHR-C075 (Okenshoji Co., Ltd., Tokyo, Japan), and 1671 (JEOL Ltd., Tokyo, Japan), and the price was around $10–20 per single grid. The micro-grids are manufactured by attaching a micro-porous support film to 200 mesh copper grids. The size of the micro-grid hole diameter is between 2.5 and 10 μm. Since the carbon film is prepared between a water–gas interface, the thickness is extremely uniform. In addition, because of membrane tension, the carbon film does not distort. 3-morpholinopropane-1-sulfonic acid (MOPS, Nacalai Tesque, Kyoto, Japan), potassium chloride (KCl, Nacalai Tesque, Kyoto, Japan), hydrochloric acid (HCl, FUJIFILM Wako Pure Chemical Corp., Osaka, Japan), and potassium hydroxide (KOH, FUJIFILM Wako Pure Chemical Corp., Osaka, Japan) were used as the electrolyte solution. The buffer solution was prepared using ultrapure water from a Milli-Q (Merck Millipore Corp., Burlington, MA, USA) at 18.2 MΩ at 25 °C. For channel current measurements of solid-state nanopores and nanopore fabrications by the DB method, we prepared chambers made from polydimethylsiloxane (PDMS; Sylgard 184, Dow Corning Toray Co., Ltd., Tokyo, Japan) in which a Ag/AgCl electrode was incorporated (Figure S1). The micro-grid was sandwiched between 0.2 mm thick polymethyl methacrylate plates (PMMA plates, Mitsubishi Chemical Corp., Tokyo Japan) with a hole in the center and fixed with glue. Then, the PMMA plate incorporating the micro-grid was sandwiched between PDMS chambers.

### 2.2. Fabrication of Nanopores by STEM EB Method

For the irradiation with a focused electron beam, Field Emission TEM (FE-TEM) (JEM-2200FS; JEOL Ltd., Tokyo, Japan) with 200 kV acceleration voltage was used. The spot diameter of the focused electron beam was set to 1.5 nm. We conducted nanopore fabrication whilst checking the image displayed on the screen of Analysis Station (JEOL Ltd., Tokyo, Japan), in which an energy dispersive spectroscopy (EDS) analysis system is linked with FE-TEM. 

### 2.3. Fabrication of Nanopores by DB Method

The DB method was performed by applying 10 or 30 V of voltage through the micro-grid fixed in the PDMS chamber using an electrochemical analyzer (ALS600DZ, BAS Inc., Tokyo, Japan) or a DC power supply (DPS-3003, CUSTOM Corp., Tokyo, Japan). We added 2 M of KCl electrolyte into both chambers and applied the voltage. While applying the voltage, we monitored the current passing through the carbon membrane using a digital multimeter (CD771, Sanwa electronic instrument). When the monitored current increased rapidly, we turned off the voltage. The pore size was calculated from the current using Hille’s equation [41]. In the DB method, we investigated the influence of pH, applied voltage, and membrane thickness on the fabrication time and pore size.

### 2.4. Channel Current Measurements and Data Analysis

For nanopores fabricated by the STEM EB method, an oxygen plasma was exposed to the micro-grids with a plasma etching system (FA-1, Samco Inc., Kyoto, Japan) to prepare a hydrophilic surface before the channel current measurements. Due to the hydrophobicity of carbon films, the plasma treatment must be first applied to increase hydrophilicity and facilitate the entry of water into the nanopores. Next, the micro-grid was incorporated into the PDMS chambers, and 1 or 0.1 M of KCl solution (10 mM MOPS, pH 7.0) was added to the chambers. Then, 2 V of potential was initially applied through the nanopore to allow passage of the electrolyte solution into the nanopore until the ion current was obtained. In the DB method, the channel currents were measured in the same solution as the nanopore fabrication (2 M KCl). Channel currents were monitored using a patch-clamp amplifier (PICO2, Tecella, Foothill Ranch, CA, USA) with a 4-kHz low-pass filter at a sampling frequency of 20 kHz. Channel current measurements were conducted at 22 ± 2 °C. Analysis of the channel signals was performed using pCLAMP ver. 10.7 (Molecular Devices, CA, USA).

## 3. Results

### 3.1. Nanopores Fabricated by the STEM EB Method

We attempted to fabricate nanopores in the carbon membrane coating on commercially available micro-grids by irradiating it with a STEM electron beam. There have been previous reports of nanopore fabrication using STEM EB irradiation of thin films of silicon materials or graphene, and precise pore size control has been achieved [19]. In contrast, there have been few reports of nanopore fabrication in thin carbon membranes as demonstrated in our experiments, and we would like to highlight the novelty of this nanopore fabrication process.

**Figure 2:**
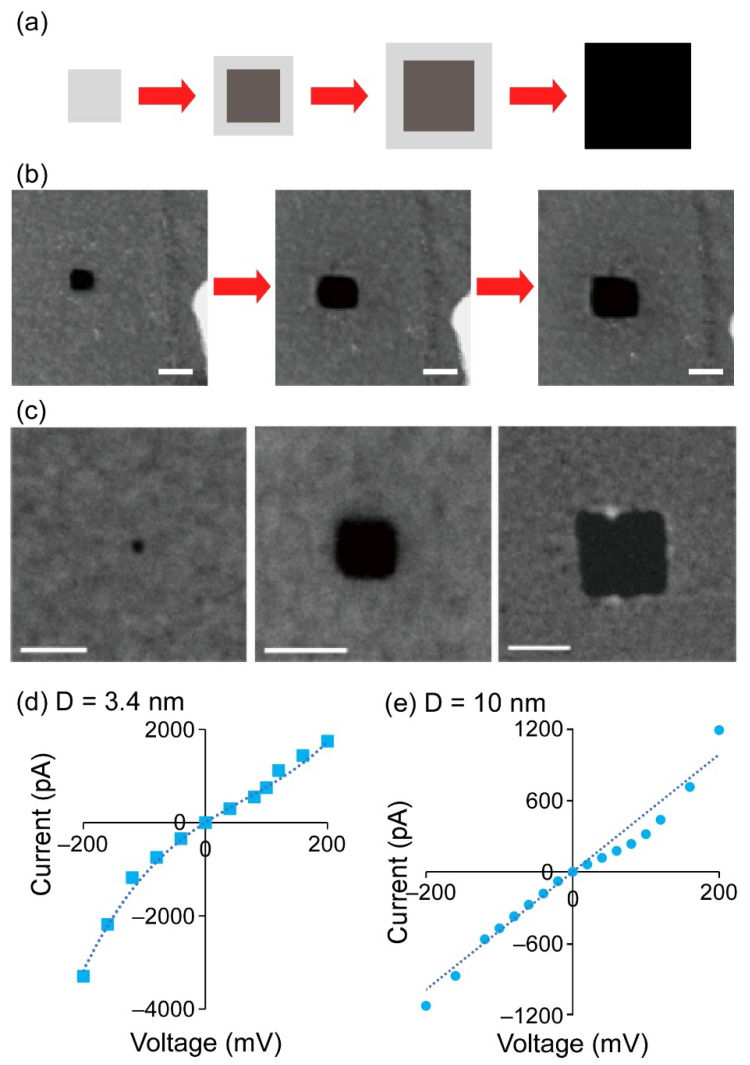
Nanopores fabricated by the STEM EB method. (**a**) The scanning pattern of a focused EB. The EB was initially irradiated over a small area to fabricate a small pore. Then, the scanning area was extended to expand the pore. (**b**) Fabrication processes of a nanopore in a carbon thin film. A small pore was expanded in size by controlling the scan area of a focused EB. The scale bars indicate 20 nm. (**c**) Three different sized nanopores: (left to right) 2.0 nm, 8.5 nm, and 45 nm in diameter. These diameters were calculated by approximating to a circle. *I-V* curves of nanopores with (**d**) 3.4 nm (in 1 M KCl solution) and (**e**) 10 nm (in 100 mM KCl solution) diameter fabricated by the STEM EB method.

First, we tried to fabricate various sizes of nanopores (>2 nm in diameter) by increasing the scanning area of the EB using the following procedures. Irradiation with the focused EB on one point of the carbon film for several tens of seconds caused a knock-on damage to the carbon, resulting in the ejection of carbon. As a result, a small pore was formed where the EB radiation was applied. Then, the focused EB was scanned in a specified area for tens of seconds up to 90 min, causing the pore to gradually expand (Figure 2a,b). This resulted in the fabrication of square-shaped nanopores and successful control of pore diameter from 2 to 135 nm (Figure 2c and Figure S2). The carbon spectrum was not detected in the nanopore region with the EDS elemental analysis. Thus, we judged that nanopores were completely open in the carbon film. The area was measured from the TEM images using the “Image J” software, and the pore diameter was calculated from the area by approximating a circle. Because the scan direction of the EB is fixed in the horizontal direction, the shape of the EB-scanned area became rectangular. As a result, square-shaped nanopores were constructed. An advantage of this STEM method is that the nanopore fabrication process can be performed while observing the STEM image. Additionally, when nanopores are fabricated with the STEM method in a free-standing SiNx membrane, the free-standing area must be restricted to around 50 μm square to prevent the breakage of the membrane. On the other hand, in the case of our TEM-grid membrane, a nanopore can be fabricated in a larger 3 mm × 3 mm carbon membrane, because there is a supporting micro-porous layer underneath the carbon thin membrane. In addition, we tried different EB irradiation patterns to reduce the fabrication time (Figure S3). However, the process detailed above (Figure 2a) was better than these two methods in terms of the pore formation efficiency.

Next, we evaluated the nanopores by obtaining the current and voltage (*I-V*) nanopore curves (Figure 2d,e and Figure S4). The *I-V* curve of the 3.4 nm nanopore shows an asymmetric shape, but that of the 10 nm nanopore does not. An asymmetric *I-V* curve indicates a conical-shaped nanopore, and such an effect of the pore geometry on the *I-V* curve is expected to be observed in small nanopores [42,43]. Conventional ssNPs fabricated by irradiating EB at the same point on SiNx thin membrane [27] provide an hourglass shape. On the other hand, we can deduce that since the STEM EB was irradiated from one side and continuously scanned, conical-shaped nanopores were fabricated, resulting in the asymmetric *I-V* curve for the smaller nanopore. By contrast, a symmetric *I-V* curve was obtained from the larger nanopore, although the nanopore owns a conical geometry. In nanopore sensing, the shape of nanopores significantly influences the sensitivity of molecular analysis because the resistance of nanopores relates to the narrowest zone of the nanopore. Our *I-V* evaluations demonstrate that the effective length of the nanopores is shorter than the thickness of the carbon thin film, suggesting that they would be useful as nanopore sensors. However, we found the larger current noise at large absolute values of the applied potential (Figure S4). Because the ratio of the membrane thickness to area is significantly large (10 nm:7 mm^2^), mechanical fluctuations in the membrane would strongly influence the current noise [44]. To improve the current noise, the self-standing membrane area should be reduced by using a micro-grid with smaller apertures.

From these results, we can see that the STEM EB method allowed us to fabricate nanopores in commercially available carbon thin films. The effective length of the nanopores should be shorter than the thickness of the film, whilst the pore size can be precisely controlled over a wide range (2–130 nm in diameter). However, we could not demonstrate nanopore sensing of DNA molecules with the nanopores. We believe that the adsorption of DNA molecules on carbon influenced the performance of nanopore sensing. In the future, we need a surface modification on carbon membranes to prevent the adsorption.

### 3.2. Nanopores Fabricated by the DB Method

Although we demonstrated successful fabrication of nanopores in commercially available carbon films with high accuracy by the STEM EB method, the STEM EB method is still labor-intensive since a FE-TEM is expensive, and its operation requires specific skills and training. Therefore, we next applied the DB method for nanopore fabrication in a carbon film to achieve easier and more rapid nanopore fabrication. To the best of our knowledge, the DB method is the simplest method to fabricate solid-state nanopores in thin films because nanopores can be fabricated by simply applying a voltage through the film in an electrolyte solution, as detailed below [19].

**Figure 3:**
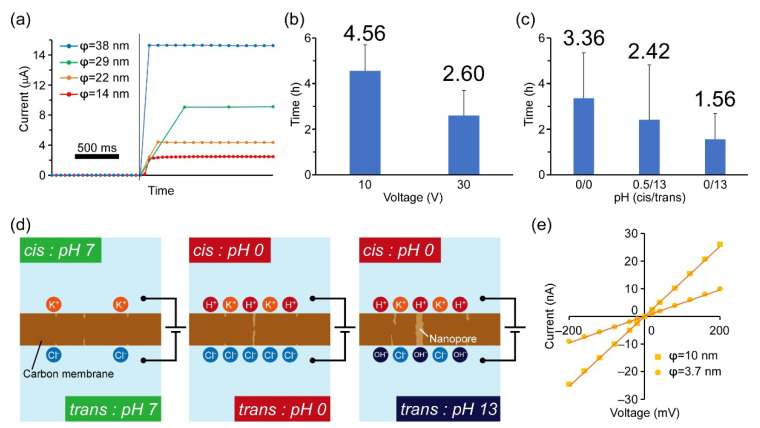
Nanopore fabrication using method 2 (DB + carbon membrane). (**a**) Current and time traces of the DB experiments with several different nanopore formations estimated from the ion currents. (**b**) The time of nanopore formation by applying different voltages (pH 0.5/0.5.) (**c**) The time of nanopore formation depending on the pH gradations; (*cis*/*trans*) means (positive/negative) application of voltages. (**d**) Schematic diagrams of the proposed mechanism for the break down by pH gradation. (**e**) *I-V* curves of the nanopores fabricated by the DB method for different pore sizes.

First, the application of a transmembrane voltage through the carbon film generates an electric field inside the carbon film and charges the interfaces with opposite ions. Then, the electric field induces bond breakage, resulting in structural defects in the film. When the structural defects accumulate, a highly localized conductive path forms, and a nanopore is constructed in the film (Figure S5) [25]. In conventional studies of nanopore fabrication in SiNx thin membranes, nanopores were fabricated when the applied electric field was larger than the dielectric breakdown strength. On the other hand, because the carbon membrane with free electrons is a conductive material, the applied transmembrane voltage cannot be determined with the same strategy used in previous works. Hence, in the DB experiments for the carbon film, we attempted to apply two different transmembrane voltages of 10 and 30 V. As a result, we obtained current increases after 4.56 ± 1.14 h and 2.60 ± 1.10 h with 10 and 30 V, respectively (Table 1 and Figure 3a,b). In addition, the current signals could be classified into several main patterns (Figure S6). The first one was a step-like current increase in which the current rapidly increased and became constant (Figure 3a and Figure S6c). Approximately 60% of the current signals appeared as this signal. In other patterns, for example, gradual increases in the currents (Figure S6a) and current decreases after an initial increase in current (Figure S6b) were obtained. The gradual current increase might indicate that multiple pores were formed [45,46]. In the DB method, structural defects potentially occur across the whole area where the transmembrane voltage is applied. Since the carbon thin film area is much larger than conventional nanopore fabrication in SiNx membranes by the DB method, the probability of multiple pore formation is much higher than when using SiNx. Signals with a decrease in the current would suggest that complete nanopores were not formed. Because of the increase of leakage currents through the membrane by several discrete structural defects, breakdown events at a single point did not ultimately occur, and nanopores were not fabricated. On the other hand, the single step-like current signals indicate the formation of a single nanopore. Therefore, when the single step-like signal was obtained, we halted the voltage a few seconds after increasing the current.

Although the formation of nanopores in the carbon film was confirmed by measuring the ion current, the fabrication times of nanopores (4.56 ± 1.14 h @ 10 V and 2.60 ± 1.1 h @ 30 V) (Table 1 and Figure 3b) were much longer than the conventionally reported nanopore fabrication in SiNx film by the DB method. Therefore, we attempted to improve the processing time of pore fabrication by changing the pH gradient between the *cis* and *trans* sides of the membrane. The *cis*/*trans* refers to positive/negative application of voltage at the membrane. As shown in a previous report [47], a pH gradient facilitates the accumulation of charged ions on the membrane surface and can shorten the fabrication time (Figure 3d). As a result of increasing the pH gradient between *cis* and *trans*, we were able to reduce the fabrication time of nanopores to 1.56 ± 1.14 h with the pH gradient (*cis/trans*) = 0/13 (Figure 3c and Table 1). By contrast, nanopores were not fabricated with a solution on the *cis* side above pH 2.2 even when the voltage was applied for 6 h. Although the fabrication time could be reduced to around 2 h, it is still much longer than in SiNx thin membranes (several minutes). This result is reasonable because carbon has free electrons (conductive material). Therefore, the required magnitude of the electric field that causes the breakdown event is much higher than for SiNx membranes. One way to reduce the fabrication time for the carbon film would be to increase the applied transmembrane voltage, as in a previous study where the fabrication time decreased exponentially with an increasing electric field [25]. However, when we applied 30 V to the carbon film with 6 nm thickness, the current increases were too rapid, and the pore size became too large. Thus, we did not increase the applied voltage by more than 30 V.

Next, we discuss the pore size. Because previous studies have reported that the shape of nanopores fabricated by the DB method can be assumed to have a cylindrical geometry [25], we calculated the pore diameter using the Hille equation [41], which is a theoretical model that uses the resistance of a cylindrical pore to ion flow, as below.

$$R=\left(l+\frac{\pi r}{2}\right)\frac{\rho }{\pi r^{2}}$$

where R is the resistivity of the pore, r is the pore radius, l is the length of the pore, and ρ is the resistivity of the electrolyte solution. In the DB method, the length of the pore is defined as the thickness of the carbon film, and the resistivity of the pore was calculated by the channel current measurements. We calculated that about 75% of nanopores were less than 40 nm (3.7 nm–38 nm) in diameter (Figure S7). In others, much larger nanopores were obtained (maximum: 1345 nm). However, we could not find the fabricated nanopores in the carbon film by TEM observation. Although the area of the thin film is usually restricted to around 50 μm square to obtain stable thin films and easily observe the nanopores, the area of the carbon film was much larger (3 mm in diameter) in this study, and therefore it was difficult to observe the nanopores by TEM because the nanopores can be constructed anywhere on the carbon thin film. Thus, we tried to classify single or multiple pore formation from current signals when opening pores. Since multiple nanopores would not be formed simultaneously, we hypothesized that a single nanopore was constructed when a step-like current increase was obtained. On the other hand, in other cases, we excluded these nanopores from the current evaluations.

Although we demonstrated successful fabrication of nanopores in the carbon film by the DB method, the control over pore size still needs to be improved in our system. In the conventional DB method, the pore size can be controlled by setting a threshold current at which to turn off the applied voltage, to prevent expansion of the existing nanopore with the applied voltage. In this study, however, the current rapidly increased by around several μA within a second and became constant, and the typical currents had irregular values (Figure 3a). Hence, we could not control the pore size by setting a threshold current value since we manually controlled the application of the transmembrane voltage. Therefore, in future research, we will build a current feedback program to control the applied voltage. In addition, even though the application of a pulsed voltage would offer control over nanopore size, the fabrication time will be much longer, or nanopores will not form since the bond breaking will not occur by being buffered with the movement of free electrons in the short term.

In addition, the concern that multiple nanopores were constructed on the carbon film should be dispelled. The use of micro-grids with a smaller aperture or the local DB technique [48] using a fine needle electrode, such as AFM tips, would improve the probability of single nanopore formation on the carbon membrane.

We next evaluated the nanopores by measuring the *I-V* curves. To compare them with the nanopores fabricated by the STEM EB method, we selected nanopores of 3.7 and 10 nm in diameter (calculated using Hille’s equation). Both *I-V* curves showed a linear relationship between the applied potential and the channel current, indicating that cylindrical-shaped nanopores were fabricated by the DB method (Figure 3e). 

The main advantage of the DB method compared with the STEM EB method is the much simpler fabrication procedure. Nanopores can be fabricated by simply applying a DC transmembrane voltage. However, the drawbacks include the reduced control over pore size and the lengthy time required. Although the pore diameters were mostly less than 40 nm, much larger nanopores were sometimes obtained and the fabrication time was more than 1.5 h for any experimental conditions. 

Finally, we discuss the comparison between the nanopores fabricated in commercially available carbon thin films and SiNx thin films (Table 2). The advantages of using carbon films are that the complex fabrication processes for the preparation of thin films are not required, leading to a simpler nanopore fabrication process. Carbon membranes can be purchased from companies or can be readily prepared on the micro-grid by transferring a floating carbon thin film. By contrast, although the SiNx thin films can also be purchased, micro-and nano-fabrication processes are required to prepare the films oneself. In the STEM EB method, there are few differences in the size controllability and the fabrication time of nanopores between the carbon films and the SiNx membranes. In the DB method, however, the fabrication time is longer and the size controllability of nanopores is lower in the flat carbon films than those of the nanopores in the SiNx thin films. Overall, the stability of the carbon film is lower than that of SiNx membranes since the area of the carbon film in a micro-grid is much larger than that of SiNx membranes (3 mm in diameter versus around 50 μm square). The carbon films were occasionally broken when manipulating the micro-grid. Although the membrane rupture was improved by removing the static electricity of the device, the larger membrane area influences the mechanical properties. If micro-grids coated by a carbon film with a much smaller surface area were commercially available, the nanopores fabricated in such a micro-grid would offer great potential for nanopore sensing. 

## 4. Conclusions

In conclusion, we propose the use of commercially available flat thin carbon film-coated micro-grids as the material for solid-state nanopores. To fabricate nanopores in the carbon membrane, we applied the STEM EB method and the DB method. We successfully fabricated solid-state nanopores in the membrane using both methods. In the STEM EB method, the pore size was precisely controlled, ranging from 2 to 135 nm in diameter, and the fabrication time was within 90 min for each nanopore. In the DB method, the nanopores can be fabricated with a much simpler experimental setup and procedure compared with the STEM EB method. In contrast, the fabrication time was longer than the STEM EB method due to the conductivity of the carbon film. In addition, although the pore diameter was less than 40 nm for 75% nanopores, precise control over pore size was not achieved due to the rapid current increase when opening the pore. We believe that nanopores fabricated in commercially available carbon thin films could be widely used in nanopore research due to the lack of requirement of micro-and nano-fabrication for thin-film preparations.

## Figures and Tables

**Table 1:** The relationship between the film thickness, applied voltage, pH, and fabrication time.

Applied Voltage (V)	pH (Cis/Trans)	Thickness of the Membrane (nm)	Fabrication Time (h)
10	0.5/0.5	6	4.56 ± 1.14
30	0.5/0.5	6	2.60 ± 1.10
10	0/0	10	3.36 ± 1.99
10	0.5/13	10	2.42 ± 2.41
10	0/13	10	1.56 ± 1.14

**Table 2:** Comparison of fabrication methods of solid-state nanopores.

Methods	Materials	Pore Size (nm)	Size Controllability	Thickness (nm)	Pre-Micro and Nano Fabrication	Fabrication Time (min)	Membrane Stability	REF.
TEM EB	Carbon film (Micro-grid)	2~135	Excellent	−10	Not required	2~90	low	This work
DB	Carbon film (Micro-grid)	3.7~1345 (75% of nanopores are less than 40 nm)	Poor	−10	Not required	90~300	low	This work
TEM EB	Si_3_N_4_	2~20	Excellent	10-	Required	0.5~	high	[23]
DB	SiNx	1~25	Good	10-	Required	−10	high	[25]

## Supplementary Materials

The following are available online at https://www.mdpi.com/article/10.3390/mi12091135/s1, Figure S1: A PDMS device for current measurements of a carbon film-coated TEM grid with a nanopore. (a) A photograph of the top view of the device. (b) A schematic illustration of the device. A TEM grid and PMMA plates are sandwiched between PDMS chambers into which Ag/AgCl reference electrodes are incorporated., Figure S2: STEM-HAADF images of the fabricated nanopores at the carbon membrane. Scale bars are 20 nm., Figure S3: Scanning patterns of the focused electron beam and TEM images of carbon membranes. (a) Initially irradiated to form a small pore and further irradiated around the small pore to form the larger nanopore. (b) Irradiated many adjacent small pores to ultimately form a large square nanopore. The scale bars indicate 20 nm., Figure S4: Raw current data of TEM nanopores for IV curve measurements., Figure S5: Mechanism of the dielectric breakdown. Ions accumulate at the surface of the membrane when a high voltage is applied, resulting in defect formation. One of the defects subsequently grows to form a through-hole of nanometer size., Figure S6: Typical results on the three different conditions of the dielectric breakdown experiment. (a) [KCl] = 2 M, Voltage = 10 V, pH (cis/trans) = (0/0), Membrane thickness = 10 nm. The typical result shows that the current gradually increases and returns to 0. (b) [KCl] = 2 M, Voltage = 10 V, pH (cis/trans) = (0/0), Membrane thickness = 10 nm. The typical result shows that the current suddenly increases and decreases. (c) [KCl] = 2 M, Voltage = 10 V, pH (cis/trans) = (0.5/13), Membrane thickness = 10 nm. The typical result shows that the current suddenly increases and plateaus., Figure S7: A histogram of the pore diameter of nanopores fabricated by the DB method. The pore diameters were mostly less than 40 nm.
