# Supplementary material for: Simple Fabrication of Solid-State Nanopores on a Carbon Film
Source: Micromachines (Basel). 2021 Sep 21;12(9):1135. doi: 10.3390/mi12091135 (PMC8469253; doi:10.3390/mi12091135)
Supplement: Supplementary file 1 [file micromachines-12-01135-s001.zip › micromachines-1351643-supplementary.pdf]

micromachines

Supplementary Materials: Simple Fabrication of Solid-state Nanopores Using A Commercially Available Material

Natsumi Takai, Kan Shoji, Tei Maki and Ryuji Kawano

Figure(s):

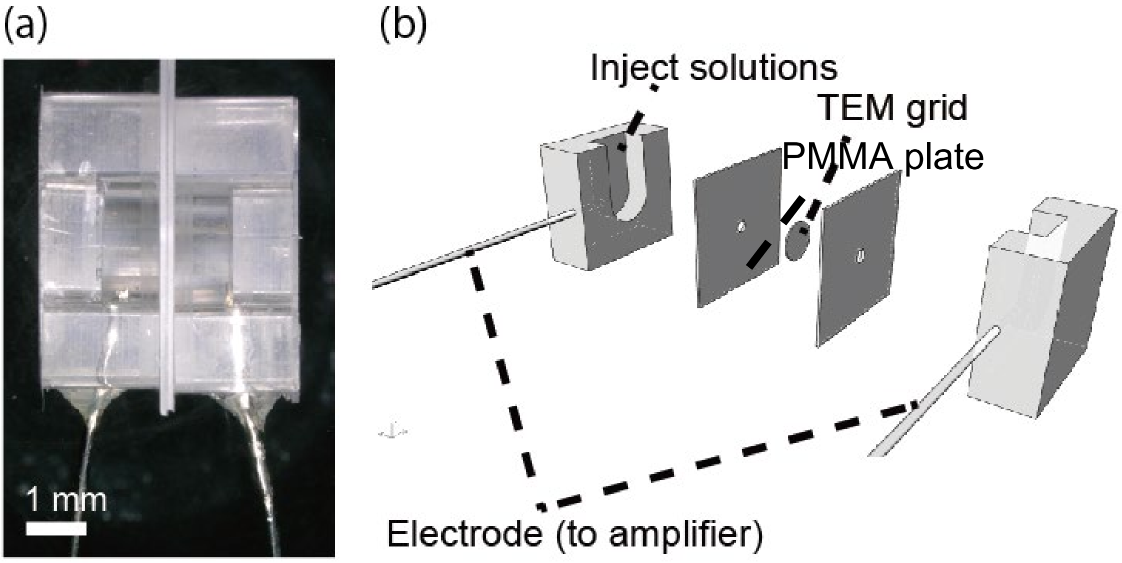

Figure S1. A PDMS device for current measurements of a carbon film-coated TEM grid with a nanopore. (**a**) A photograph of the top view of the device. (**b**) A schematic illustration of the device. A TEM grid and PMMA plates are sandwiched between PDMS chambers into which Ag/AgCl reference electrodes are incorporated.

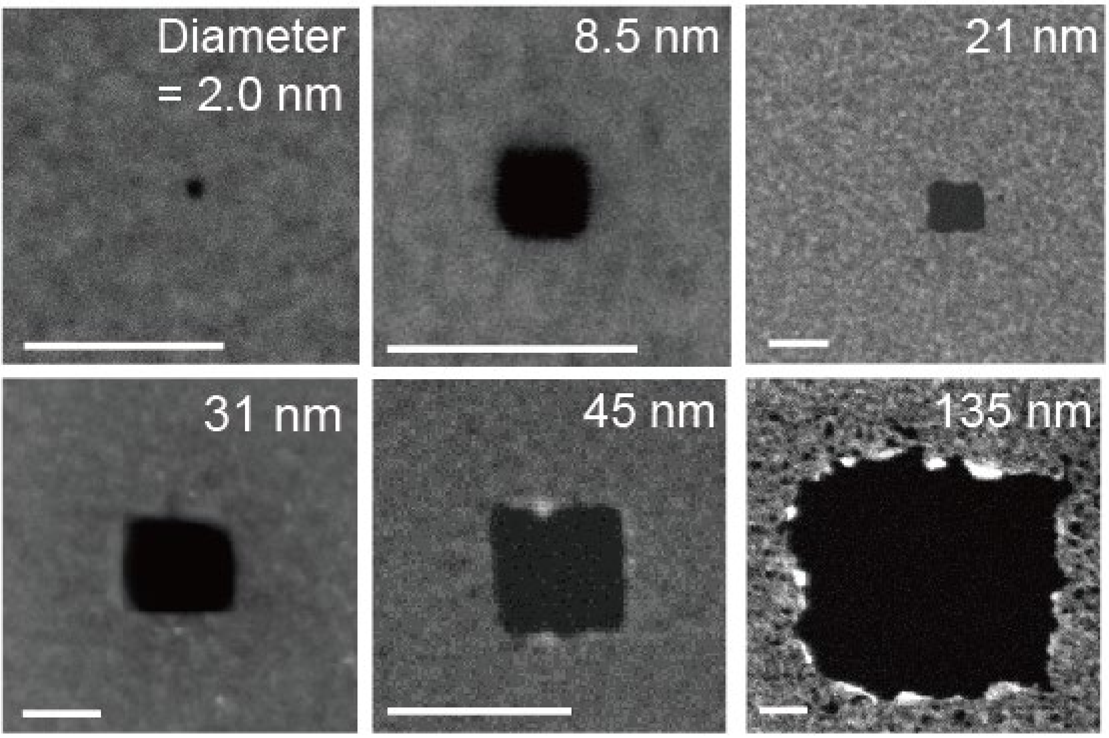

Figure S2. STEM-HAADF images of the fabricated nanopores at the carbon membrane. Scale bars are 20 nm.

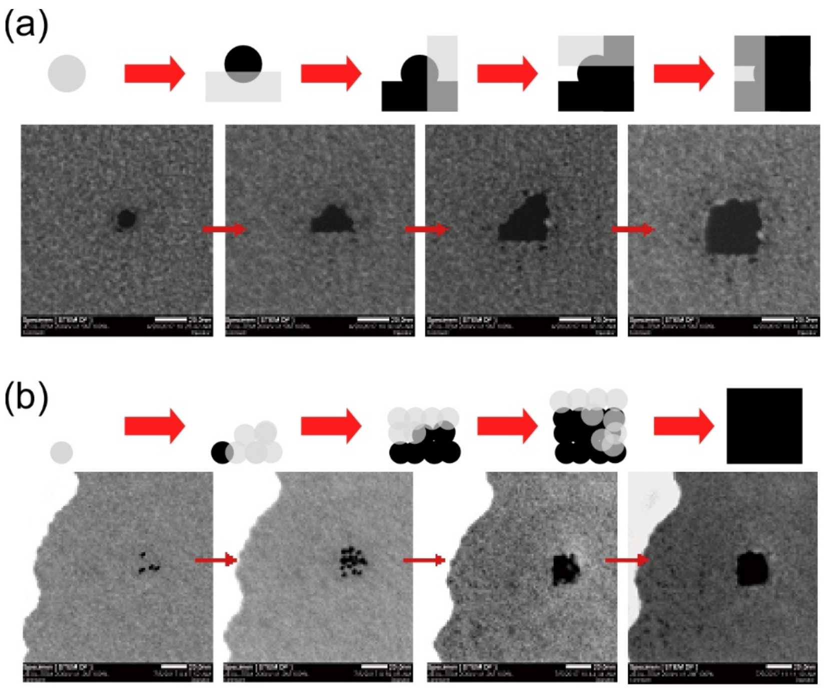

Figure S3. Scanning patterns of the focused electron beam and TEM images of carbon membranes. (**a**) Initially irradiated to form a small pore and further irradiated around the small pore to form the larger nanopore. (**b**) Irradiated many adjacent small pores to ultimately form a large square nanopore. The scale bars indicate 20 nm.

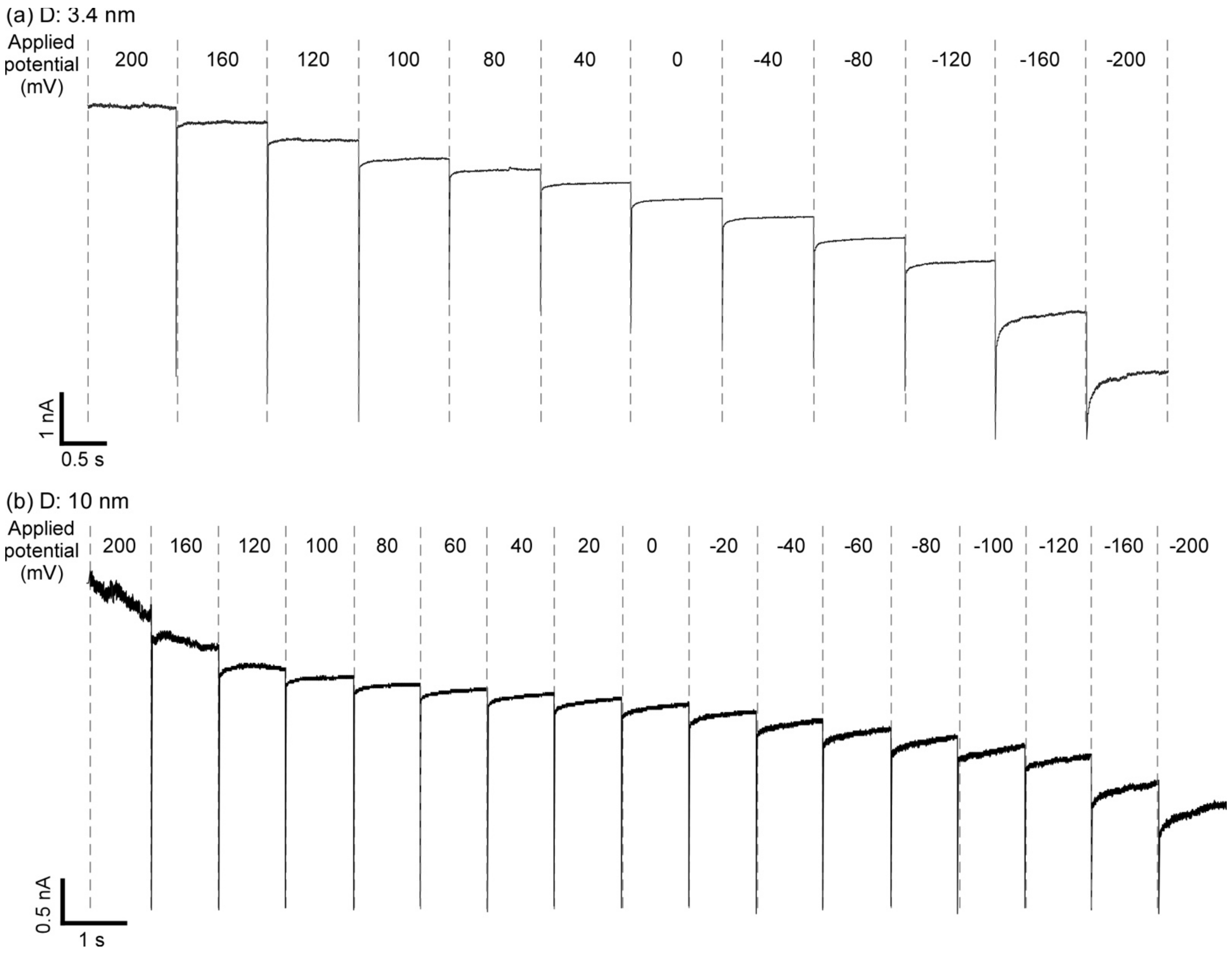

Figure S4. Raw current data of TEM nanopores for IV curve measurements. When the absolute values of applied potentials are larger than 100 mV, the current noise became larger.

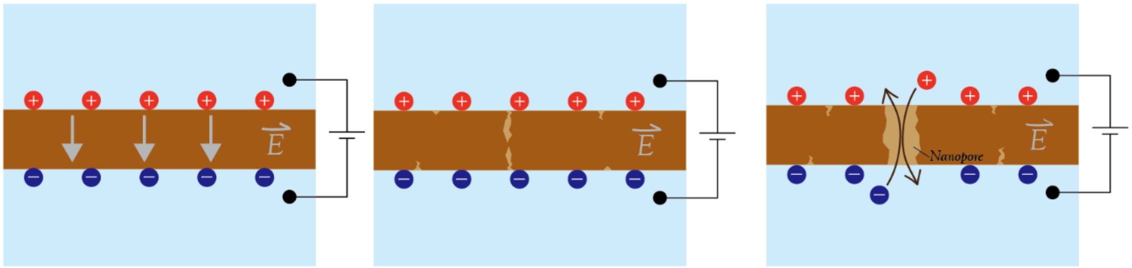

Figure S5. Mechanism of the dielectric breakdown. Ions accumulate at the surface of the membrane when a high voltage is applied, resulting in defect formation. One of the defects subsequently grows to form a through-hole of nanometer size.

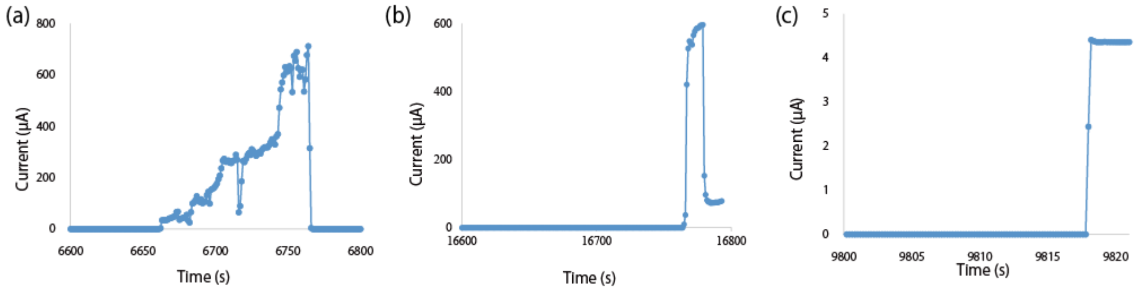

Figure S6. Typical results on the three different conditions of the dielectric breakdown experiment. (a) [KCl] =2 M, Voltage = 10 V, pH (cis/trans) = (0/0), Membrane thickness = 10 nm. The typical result shows that the current gradually increases and returns to 0. (b) [KCl] =2 M, Voltage = 10 V, pH (cis/trans) = (0/0), Membrane thickness = 10 nm. The typical result shows that the current suddenly increases and decreases. (c) [KCl] =2 M, Voltage = 10 V, pH (cis/trans) = (0.5/13), Membrane thickness = 10 nm. The typical result shows that the current suddenly increases and plateaus.

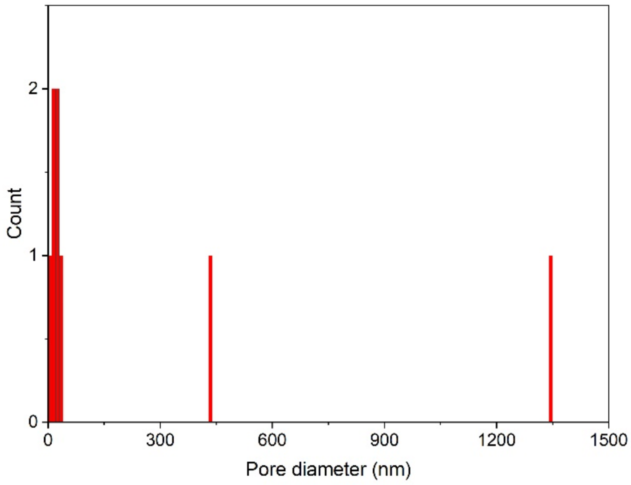

Figure S7. A histogram of the pore diameter of nanopores fabricated by the DB method. The pore diameters were mostly less than 40 nm.
